# 肺结节术前定位的研究进展

**DOI:** 10.3779/j.issn.1009-3419.2025.101.07

**Published:** 2025-05-20

**Authors:** Jialong CHEN, Lei ZHOU, Lingling QIN, Chunlai LIU

**Affiliations:** 441000 襄阳，湖北医药学院附属襄阳市第一人民医院心胸外科; Department of Cardiothoracic Surgery, Xiangyang No.1 People’s Hospital, Hubei University of Medicine, Xiangyang 441000, China

**Keywords:** 肺结节, 计算机断层扫描, 电视辅助胸腔镜技术, Pulmonary nodule, Computed tomography, Video-assisted thoracic surgery

## Abstract

近年来，随着计算机断层扫描（computed tomography, CT）筛查技术的广泛应用，肺结节检出率明显上升。作为肺癌早期诊断的重要指标，电视辅助胸腔镜手术（video-assisted thoracic surgery, VATS）是肺结节治疗的首选方案。研究表明，精准的术前定位能显著提高手术成功率（降低中转开胸率）、减少并发症并缩短手术时间。本文系统综述了六大核心定位技术：经皮穿刺辅助定位、电磁导航支气管镜（electromagnetic navigation bronchoscopy, ENB）定位、3D打印辅助定位、“流域分析”法定位、机器人辅助导航系统定位及混合现实（mixed reality, MR）定位。通过对比分析各技术的操作原理、有效性、安全性及临床适用性，本文旨在为优化肺结节管理的临床决策提供循证医学依据。

## Abstract (en)

In recent years, the widespread application of chest computed tomography (CT) screening has led to a significant increase in the detection rate of pulmonary nodules. As a critical diagnostic tool for early-stage lung cancer, video-assisted thoracic surgery (VATS) has emerged as the preferred therapeutic approach for pulmonary nodules. Clinical evidence demonstrates that precise preoperative localization significantly enhances surgical success rates (reducing conversion to thoracotomy), minimizes complications, and shortens operation time. This comprehensive review systematically evaluates six cutting-edge localization techniques: percutaneous puncture-assisted localization, electromagnetic navigation bronchoscopy (ENB) localization, 3D-printed auxiliary localization, basin-analysis-based localization, robotic navigation system localization, and mixed reality (MR)-guided localization. By critically analyzing their operational principles, efficacy, safety profiles, and clinical applicability, this paper aims to provide evidence-based recommendations for optimizing clinical decision-making in pulmonary nodule management.

肺癌是中国及世界范围内最常见的恶性肿瘤之一，其致死率也位居前列，发病率和死亡率在逐渐上升，约占所有癌症新发病例的12.4%。国际癌症研究机构（International Agency for Research on Cancer, IARC）2024年发表的数据^[1]^显示，每年全球新增肺癌病例约为250万例，在癌症发病率与死亡率方面均居首位，其中男性肺癌发病率高于女性。根据国家癌症中心2024年公布的数据^[2]^，2022年我国新增肺癌病例约为106.06万例，居全国各类癌症之首，城市地区的肺癌发病率通常高于农村地区，这可能与空气污染、吸烟习惯和工业化有关。每年约73.33万人因肺癌离世，占癌症相关死亡人数的28.4%，肺癌的高死亡率与确诊时多处于晚期密切相关。尽管肺癌治疗手段不断进步，但我国肺癌患者的5年生存率仍较低，仅约20%。

肺结节是指在胸部影像学检查中发现的直径<3 cm的局部性肺部阴影，通常在计算机断层扫描（computed tomography, CT）中被识别。在影像学中，肺结节主要有实性结节、部分实性结节和磨玻璃结节三种。实性结节通常具有较高的恶性潜能，而磨玻璃结节则可能是早期肺癌的表现，尤其是当其直径超过10 mm时，其恶性概率可高达50%。此外，肺结节的大小、形态、边缘特征及其生长速度等因素均可影响其恶性风险的评估。对于<8 mm的结节，通常采取观察和定期随访的策略，而对于≥8 mm的结节，则可能需要进行更积极的干预，如活检或手术切除。根据指南，肺结节的管理应结合患者的风险因素、影像学特征及临床判断来综合考虑^[3-5]^。

肺结节的术前定位在电视辅助胸腔镜手术（video-assisted thoracic surgery, VATS）中具有重要的临床意义。许多肺结节在手术时可能无法通过触诊或视觉直接识别，术前定位技术的应用可以显著提高结节的识别率，降低转为开胸手术的风险^[6,7]^。肺结节的术前定位不仅提高了手术的成功率，还为患者提供了更为安全和有效的治疗方案。随着影像学技术的不断进步，术前定位技术的应用将更加广泛。本文目的旨在归纳肺结节定位的新旧方法及各自优缺点。

## 1 常见的传统术前定位方法

### 1.1 带钩金属丝定位法（Hook-wire）

CT引导下经皮穿刺钩丝定位是肺结节术前定位的经典方法。Yang等^[8]^对150例患者的研究显示，定位组（*n*=50）手术时间、出血量及住院时间均显著低于对照组，但存在8例无症状气胸及2例钩丝移位并发症。Park等^[9]^报道113例中4例钩丝移位，气胸发生率为23.0%。尽管操作简便，但钩丝移位率较高（约8%），且可能引发胸膜反应。该研究结果显示，CT引导下的钩丝定位技术，对于小肺结节的定位具有积极作用，不仅能够减少术中出血量，还能缩短手术时间与住院时长，降低中转开胸率。但该技术存在钩丝位移和脱离率较高、引发明显疼痛以及空气栓塞风险等缺点。尽管如此，因其操作相对简便，在临床上应用较为广泛。

### 1.2 微弹簧圈定位法

弹簧圈作为微创介入手术中常用的器械，现也应用于肺结节的术前定位。术前经皮穿刺将微型弹簧圈一端植入肺结节附近，另一端盘曲于胸膜表面，从而准确定位结节。Li等^[10]^对221例患者的对比研究发现，弹簧圈定位成功率为91.4%，虽低于定位针（98.6%），但避免了胸壁导丝残留。李等^[11]^的回顾性分析表明，定位组成功率为100%，术后并发症率（14.0%）显著低于未定位组（24.0%）。

### 1.3 医学生物胶定位

在影像引导下，经皮将医用生物胶注入贴近肺结节所在的位置，生物胶会快速凝固，形成一个明确的标记点帮助术者在术中更精确地识别病灶位置。Wang等^[12]^对154例术前定位患者进行分析，医用胶定位组（*n*=101）成功率为100%，钩丝定位组（*n*=53）成功率为98.1%，并发症方面两者无显著差异。Wang等^[13]^使用动物实验优化配比（医用胶:ICG=4:3），显著提升标记清晰度。该技术具有无创优势，但存在刺激性咳嗽风险，临床多联合其他方法使用。

### 1.4 其他注射型定位

临床上除医用生物胶外，还有如碘油、亚甲蓝、吲哚菁绿等也常用于定位。碘油因扩散范围小、信号持久被广泛采用；亚甲蓝则因弥散速度快，需严格控制注射时机，Rho等^[14]^将吲哚菁绿溶液和脂醇（二碘化油）组成的荧光碘化乳剂在CT透视引导下，将3种不同比例混合的乳剂分别注入3只家兔的共15个肺叶，随后完成开胸手术切除家兔肺叶，分析乳剂在肺叶表面的分布与信号强度。并且使用该乳剂对24名受试者的共29个肺结节进行术前定位分析。结果显示10% ICG与90%脂醇混合的乳状液在荧光显微镜下分布最为均匀，信号强度也最高，在兔肺中的一致性也最佳。所有类型的乳状液注射都能很好地定位在目标结节周围，并且未出现手术相关并发症。Chu等^[15]^对比显示，钩丝定位的气胸风险是亚甲蓝的5倍，王等^[16]^创新性联合自体血与ICG，使染料弥散率降低37%，定位成功率提升至86.7%。此外，宋等^[17]^设计了一种双腔同步注射器用于肺结节定位，此装置可同时注射出四氧化三铁海藻酸钠磁流体和10%葡萄糖酸钙溶液，两种溶液接触后会立即形成凝胶样结节，手术前通过胸部CT的引导，将双腔同步注射器的穿刺针针头经皮穿刺至肺小结节附近，而后在术中通过寻踪磁体（径向充磁的永磁材料N45钕铁硼，Nd-Fe-B）在肺表面探寻，寻踪磁体和磁凝胶之间会产生磁力相互吸引，使得肺小结节附近肺组织突出肺表面，从而实现定位肺结节的作用，但此方法目前只在动物实验中被应用，尚未在临床得到实施，其优缺点还需大量的临床数据去探索。

## 2 新型定位方法

### 2.1 电磁导航支气管镜（electromagnetic navigation bronchoscopy, ENB）定位

ENB是一种结合影像导航和支气管镜技术的微创方法，术前通过高分辨率胸部CT生成3D支气管树模型，标定结节位置并在导航系统中设计一条从气管入口到目标结节的“虚拟路径”，术中患者躺在电磁导航床上，胸腔上方放置电磁场发生器，产生低强度交变磁场，术者将带有电磁传感器的导管（或定位探头）通过鼻腔/口腔插入支气管镜工作通道，在电磁场中实时显示位置和方向，根据术前设计的路径逐步引导导管深入支气管，最后到达结节附近定位结节^[18]^。Wang等^[19]^通过ENB标记了25例患者共28个肺结节，其中23个结节被成功切除，未出现定位相关的并发症；Taton等^[20]^证实ENB联合冷冻活检在≤2 cm结节中具有更高诊断效能。ENB在肺结节的定位与诊断方面优势十分明显，但主要局限在于依赖支气管解剖结构，对周围型结节定位困难，其次操作难度和定位技术成本高，不利于临床推广。而且，ENB通过支气管走行定位肺结节，对于非支气管附件区域的肺结节无法做到精准定位，因此目前仍处于临床探索阶段^[21]^。

### 2.2 混合现实（mixed reality, MR）引导

MR技术通过三维模型与患者体表实时融合实现可视化定位。有研究^[22]^对3例患者进行了混合现实技术定位肺结节，最后混合显示工具复现了所有病例的术前CT解剖结构，结节定位误差<3 mm，未发生定位相关并发症。Xin等^[23]^在一项动物模型研究中评估了MR引导的肺结节定位的可行性和安全性，结果显示该方法的定位精度为（5.71±2.59）mm，定位时间为（8.07±1.44）min，无明显定位相关并发症。MR引导定位肺结节代表了精准医学与数字外科的前沿结合，尤其在微小结节展现出独特价值，该技术具有无创、可预演手术操作的优势，但设备成本高昂且依赖高质量CT数据，目前多处于临床试验阶段。

### 2.3 机器人辅助导航系统定位

机器人辅助导航系统定位肺结节是一种结合了机器人技术与医学影像引导的创新方法。Guo等^[24]^利用机器人辅助CT引导定位了599例患者的共654个肺结节，并与接受传统手动定位的90例患者进行对比，在手术准确性方面，机器人组定位成功率为96.64%（632/654），平均定位时间（22.85±10.27）min，定位锚爪脱落2例（0.31%），平均定位偏差（5.01±3.45）mm。机器人组并发症主要包括气胸（27.21%）、肺实质出血（33.94%）、胸膜反应（0.50%）和肋间血管出血（0.83%）。气胸发生率显著低于传统手动组（27.21% *vs* 43.33%）。手术结果上，所有定位成功的结节均成功切除，机器人组VATS手术时间显著缩短，尤其是肺段切除术[（93.61±35.72）vs （167.50±40.70）min]。机器人辅助CT引导下经皮肺结节钩针定位是一种安全、有效的方法，尤其适合经验不足的介入医师。该方法显著降低了气胸发生率，缩短了VATS手术时间。但机器人系统价格昂贵，普及受限；在技术方面，系统故障或软件错误可能影响定位。

### 2.4 3D打印辅助定位

3D打印技术于1981年首次被报道。在医疗领域，利用3D打印辅助定位系统定位肺结节的方法有3种，最常见的是3D打印模板导航辅助经皮穿刺定位，3D打印模板导航利用3D打印技术制作经皮穿刺定位板，在手术前将其附着在患者胸壁上，引导针头沿预定的路径插入，定位肺结节。与CT引导下的穿刺定位相比，该定位方法简化了穿刺定位程序，降低了辐射剂量。Zhang等^[25]^纳入16例患者，通过模板引导定位18个结节，肺结节定位成功率为100%，中位定位时间13 min，并发症方面除了2例无症状气胸外，没有其他并发症发生。有研究^[26]^通过3D打印技术为16例肺结节手术患者进行定位，成功率为94.1%，3D辅助经皮定位成功率高，并发症少；另一种3D打印模板辅助定位是通过CT图像数据进行数字重建和3D打印，定制出真人大小的模拟肺结节定位模型，术者参照模型利用肺各解剖标志进行术中定位。Li等^[27]^对通过定制化肺模型实现12个结节全切，术后残肺扩张良好；第三种3D打印定位方法由Tang等^[28]^报道，研究者在12例患者术前通过CT设计出一种柔性的可放置于胸腔内的3D模型（以第一肋骨、脊柱及胸锁关节突当作模型的稳定基础，而后根据CT上显示的结节位于肺表面投影的位置，在模型上呈现），术中通过肋间切口将3D模型放入胸腔并按照上述3处骨性标志对应放置，在模型上预留的结节处用亚甲蓝染色，最后切除结节，此12例患者全部定位成功，并无明显并发症。局限性方面，主要包括打印成本高及小结节建模精度问题。

### 2.5 “流域分析”法定位

流域是指一个水系的干流和支流所流经的整个区域，肺动静脉在肺组织内的分布也与此类似。基于肺血管灌注原理，通过阻断目标动脉实现功能区定位。即根据术前3D重建分析，明确结节所属流域的目标靶段肺动脉，术中使用阻断带将靶动脉暂时阻断，外周注射定位染料精准定位流域内/外的肺结节。Chu等^[29]^对26例患者进行此定位，其中有25例成功定位并完成肺楔形手术。张等^[30]^对23例患者行术前“流域分析”定位后，成功对全部患者行手术切除，手术过程顺利，术后有3例并发症出现，均与手术操作无关。由于肺动脉解剖结构复杂且部分患者肺裂发育不全，导致此方法临床应用受限，Huang等^[31]^创新性提出静脉分水岭定位法（V-WALM，基于3D静脉重建模拟目标静脉区域，术中阻断靶静脉后注射定位染料），并与传统CT引导定位进行优劣对比，该研究采用回顾性队列研究分别对比了V-WALM组（*n*=50）与CT引导定位组（*n*=50）两组患者的定位成功率、手术时间、手术相关并发症与平均住院时间等，结果V-WALM组定位成功率为94%（47/50），CT组为90%（45/50）；V-WALM组平均手术时间为（95.5±26.4）min，CT组为（94.3±37.5）min；并发症方面，V-WALM组气胸发生率为12%，CT组为16%，两组出血量、引流时间及住院时间均无显著差异。该项研究显示表面V-WALM定位法在手术成功率上与CT引导定位相当，安全性上可有效避免穿刺相关并发症，尤其适用于肺上叶、尖段、舌段等解剖结构复杂的区域，简化了手术流程。该定位方式对患者创伤小，并发症少。局限性在于技术层面，荧光胸腔镜尚未在基层医院广泛使用，且术中阻断血管使得血管损伤的风险增大，对于肺实质深部或血管分布密集区域的结节定位效果可能下降。

## 3 总结与展望

随着肺结节术前定位技术的不断进步，未来的发展方向主要集中在技术创新和应用的多样化上。近年来，MR技术的引导为肺结节的术前定位提供了新的可能性。此外，机器人辅助导航系统的开发也为肺结节的定位提供了新的解决方案。

相比之下，传统定位方法（微线圈/钩针/染料注射）优点在于成本低、快速且广泛适用，缺点在于有侵入性，易出现穿刺相关并发症，定位不持久。微小结节的定位效果不理想等，适用于浅表、靠近胸膜的实性结节。ENB定位的优点在于高精准性、微创、多功能性，其定位误差小且无需穿刺，避免了穿刺并发症的发生，缺点是术前需要CT扫描加上术中实时导航，增加了高辐射暴露的风险，其次是成本高昂，对复杂解剖结构有局限性，适用于深部或周围性结节，尤其是有气道相通的病灶，以及需术中实时导航的复杂病例。MR引导定位的优点在于非侵入性、直观可视化以及动态模拟（可预演肺萎陷后结节位移，优化切除范围），缺点是依赖专业设备、操作学习曲线长，精准度受到术前CT数据质量的限制，适用于浅表或中等深度结节，在教学场景中也有一定适用价值。机器人辅助CT定位的优点在于超高精度、多模态融合（可集成荧光染色、超声等多模态影像）并且支持远程操作，缺点是高成本（机器人设备及维护费用极高），适用于经验不足的年轻医生，以及解剖结构复杂的病例。3D打印模型定位的优点是触觉直观（术者可通过触摸模型预判结节位置和周围结构）、教学优势（3D解剖展示清晰，适合复杂病例讨论），缺点是耗时耗财、对于小结节的打印效果差，适用于巨大或复杂解剖结构的结节定位，以及医学教育或术前多学科讨论。流域分析法定位优点是功能导向（结合肺功能评估，避免过度切除健康肺组织）、无创性，缺点是依赖特定软件、计算复杂（处理时间较长）且准确性存在一定争议，适用于需要精准切除的肺结节，以及肺功能受限患者的保留肺实质手术。

综上所述，肺结节术前定位的未来研究方向可能集中于：（1）多模式融合定位（如MR+ENB）；（2）人工智能辅助决策系统；（3）新型生物标志物的开发。随着技术迭代，肺结节定位将朝着更精准、微创和个体化的方向发展。
